# Bibliography

**Published:** 1920-02

**Authors:** 


### BIBLIOGRAPHY

Every-day Mouth Hygiene. This is a little book written by Dr. Joseph Head, of Philadelphia, and published by W. B. Saunders Co. It is intended only to give instruction in the mouth toilet to be used to prevent the accumulation of extraneous deposits on the teeth. It is a very good exposition of a rational method. There are some statements with which we would differ, but they are matters of personal opinion. He advises a small toothbrush with very short bristles, which perhaps is proper for the suggestions he makes for its use. We prefer a small brush as to length and width with medium long bristles set in large bunches of cone form at the free ends of the bunches. We also would have more spaces between the bunches to permit the bristles to pass between the teeth and not be bent down in use. We would also advise care in the use of the floss; it is very useful, but may do much harm. It's a good book to recommend to patients. It is well printed and attractively illustrated. It is a safer book than most that have been heretofore written.

---

Systematic Development of X-Ray Plates and Films, by Lehman Wendell, is a small book just published by the C. V. Mosby Co., of St. Louis. The author has endeavored to acquaint the dental radiographer with the art of developing his negatives so as to produce the most accurate interpretation. Every one knows how often his film or plate can not be read satisfactorily though it seemingly has covered the desired area in exposure. The trouble in most cases is that the operator has not studied the photographic art sufficiently. He may know his technique and possess a reliable machine, but is either ignorant of his chemistry or careless in using his knowledge. The best radiographs we ever saw were produced by a well-known dentist who had spent much time in the study of amateur photography and had mastered the use of his chemicals. Dr. Wendell's book is excellent for a beginner, but we wish he had given us a more comprehensive treatment along the line of scientific photography as applied to radiography by the dentist who wants to do his own work more satisfactorily than he can have it done by an office assistant or commercial radiologist. Most dentists do not care to do their own developing because of the injury to their hands, or they don't want the bother of keeping their chemicals in proper working condition, consequently their work will always be unsatisfactory. This little book should stimulate better photography and create a new interest in a most important branch of dental radiography.

---

Modern Dentistry for the Laity. This is the third edition of this book by Alfred A. Crocker. This edition has been fully revised and brought up to date. The demand for the book shows a remarkable development in this phase of dentistry. Many of the largest manufacturing and commercial organizations in the country are making dentistry an important item in the welfare of their employes. The demand, as in other departments of dentistry, has long since overrun the supply of dentists and put a check on the hopes and enthusiasm of the advocates of industrial dentistry. The book is well printed and a credit to the *Register* press.

